# Job Stress, Psychological Distress, and Menstruation‐Related Symptoms in Female Workers: A Cross‐Sectional Study

**DOI:** 10.1111/1471-0528.18153

**Published:** 2025-03-28

**Authors:** Natsu Sasaki, Kazuhiro Watanabe, Miho Egawa, Yuka Ito, Yoshiaki Kanamori, Rikako Tsuji, Mako Iida, Daisuke Nishi

**Affiliations:** ^1^ Department of Mental Health Graduate School of Medicine, the University of Tokyo Tokyo Japan; ^2^ Department of Public Health Kitasato University School of Medicine Sagamihara Japan; ^3^ Department of Gynecology and Obstetrics Kyoto University Graduate School of Medicine Kyoto Japan; ^4^ Department of Psychiatric Nursing Graduate School of Medicine, the University of Tokyo Tokyo Japan

**Keywords:** gynaecology, health promotion, menstruation, premenstrual symptoms, women's health

## Abstract

**Objective:**

This study examined the relative contributions of mediation and interaction by psychological distress to the association between job stressors and menstruation‐related symptoms.

**Design:**

A cross‐sectional study.

**Setting:**

Online survey in August 2023.

**Population:**

Japanese full‐time female employees aged 20–44 not taking contraceptives.

**Methods:**

Four‐way decomposition analysis was used to estimate the relative contributions of psychological distress (mediation and interaction) to the potential pathways from job stressors and menstruation‐related symptoms.

**Main Outcome Measures:**

The Menstrual Distress Questionnaire assessed menstruation‐related symptoms before and during menstruation.

**Results:**

Of 1818 participants, 995 (54.7%) demonstrated severe menstruation‐related symptoms. Multivariable logistic regression showed that high job demands (OR = 1.50, 95% CI: 1.27–1.84) and low coworker support (OR = 1.57, 95% CI: 1.22–2.02) were associated with menstruation‐related symptoms whereas job control and supervisor support were not. Relative excess risks due to interaction with psychological distress indicated were positive and large in coworker support (RERI = 1.04, 95% CI: −0.34–2.41). In the four‐way decomposition analysis, the pure indirect effect (48.4%) and controlled direct effect (37.8%) accounted for a large part in job demands. In contrast, the proportion attributable interaction (44.1%) dominated the total effect and controlled direct effect (15.6%) accounted for fewer effects in coworker support.

**Conclusions:**

Psychological distress appeared to be an important determinant of menstruation‐related symptoms in relation to job stressors. This study suggested that reducing job demands and improving coworker support accompanied with mental health care can mitigate the adverse effect of job stressors on menstruation‐related symptoms.

## Abstract 2

**Linked article:** This article is commented on by Bone pp. 1452–1453 in this issue. To view this article visit https://doi.org/10.1111/1471‐0528.18231.

## 1 Introduction

Menstruation‐related symptoms are one of the crucial burdens in women's health, encompassing a variety of physical and psychological experiences that women may encounter before and during their menstrual cycle. Almost three in four women suffer from menstruation‐related symptoms [1]. The annual economic burden extrapolated in Japan was estimated at 8.6 billion US dollars, mainly due to work productivity loss [2]. Severe premenstrual mood symptoms can lead to suicide [3] and depression [4]. Therefore, understanding the epidemiological path of developing the symptoms is crucial to decrease the burdens of menstruation‐related symptoms among female workers.

**FIGURE 1:**
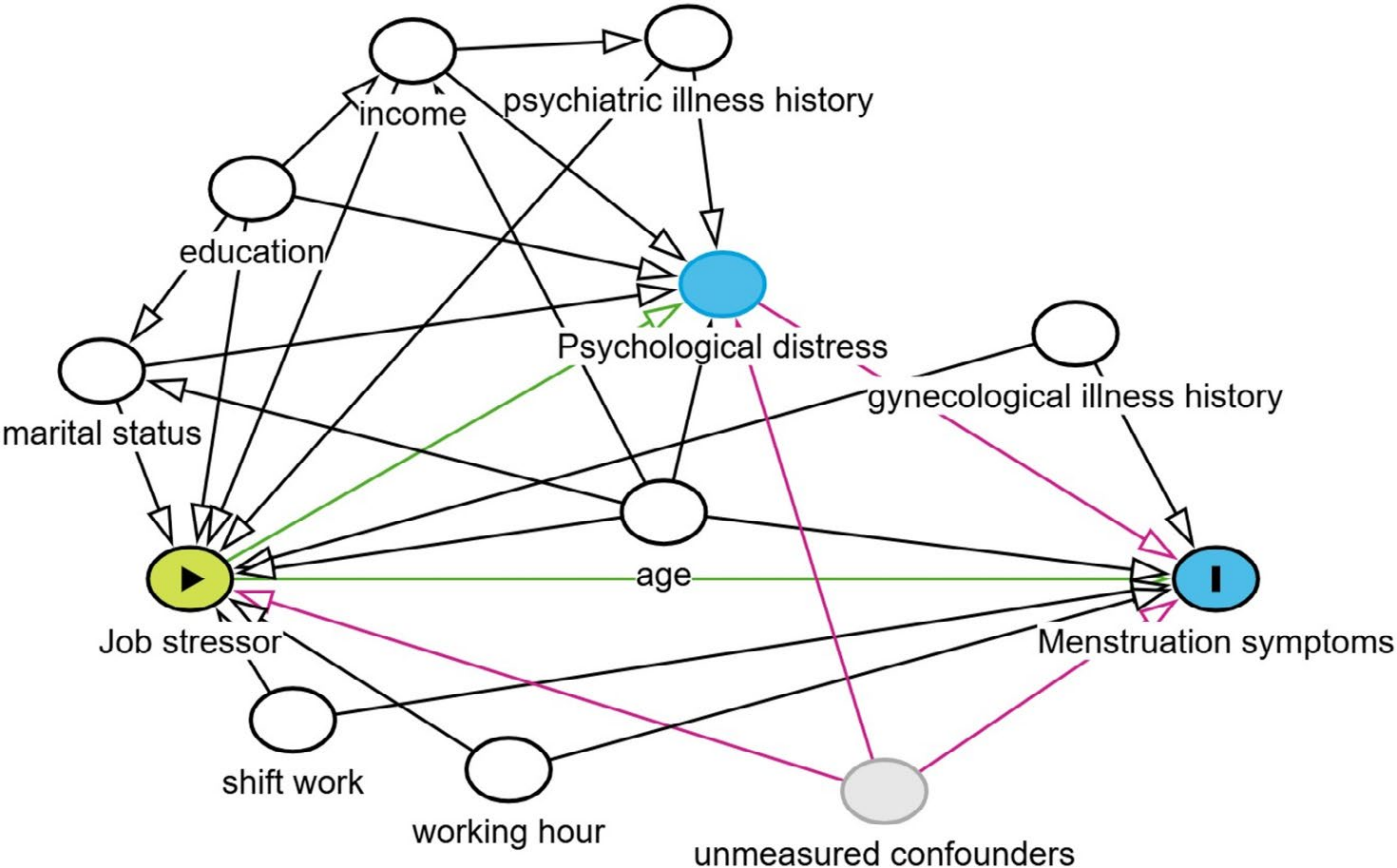
Directed acyclic graph.

Job stressors have a relationship with menstruation [5, 6]. For example, job demands, work pressure, less autonomy and less variety in work were reported to be associated with premenstrual syndrome [7]. Also, job stressors are well‐known predictors of workers' mental health [8]. Mental health may have a potential role in the association between job stressors and menstruation outcomes. Poor mental health heightens sensitivity to intense hormonal fluctuations and causes many symptoms before and during menstruation [9]. A potential directed acyclic graph is presented in Figure 1. However, these associations are complex and multifaceted; few studies have investigated the role of mental health in the link between job stressors and menstruation. To address this gap, it is essential to explore how mental health mediates and interacts with job stressors to influence menstruation‐related symptoms.

There are two ways by which mental health can link job stressors to menstruation‐related symptoms: (i) mediation and (ii) interaction. The mediation pathway hypothesizes that high job stressors lead to poor mental health, which, in turn, affects menstruation‐related symptoms [10]. Otherwise, the interaction pathway hypothesizes that poor mental health exaggerates the adverse impacts of job stressors on menstruation‐related symptoms. The mediation and interaction pathways are both likely to be possible. Yet, conventional mediation analysis ignores an exposure‐mediator interaction, resulting in biased and potentially misleading results [11]. This means the existing mediation analysis does not consider that job stressors cause poor mental health. Since the levels of job stressors can worsen mental health, the mediating effect of mental health on menstruation‐related symptoms can become intense. In this case, a new strategy should be taken if exposures and mediation factors are not independent. Nonetheless, few studies have thoroughly examined the mechanisms linking job stressors and menstruation‐related symptoms, simultaneously considering both mediation and interaction pathways.

**FIGURE 2:**
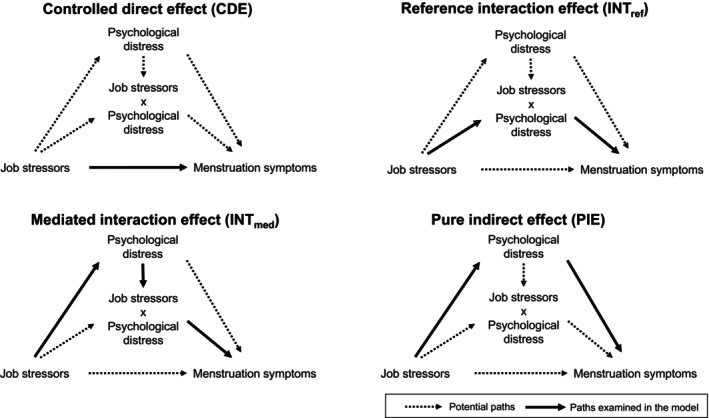
Causal diagram representing four‐way decomposition.

Four‐way decomposition analysis can estimate the relative contributions of mediation and interaction factors to the potential pathways from exposure and outcome [12]. It has a strength in considering the mediated interaction effect, that job stressors cause poor mental health, and the interaction between job stressors and mental health (i.e., job stressors make menstruation‐related symptoms worse only in cases of poor mental health). Analyses using the model that accounts for both mediating and interactive effects can provide more accurate epidemiological insights into the factors associated with menstruation‐related symptoms among female workers (Figure 2).

This study aimed to examine the relative contributions of mediation and interaction by psychological distress to the association between job stressors and menstruation‐related symptoms among Japanese pre‐conceptional female workers. A four‐way decomposition approach, which is a causal inference method that unifies the analysis of mediation and interaction, allows us to investigate (i) the direct effect of job stressors on menstruation‐related symptoms, (ii) the underlying mechanisms from job stressors to menstruation‐related symptoms through mental health, (iii) the impact of interaction between job stressors and mental health on menstruation‐related symptoms and (iv) the impact of interactions between job stressors and poor mental health caused by job stressors on menstruation‐related symptoms.

## 2 Methods

### 2.1 Study Design and Setting

This cross‐sectional study used data from an online survey of Japanese full‐time female employees in October 2023 (*N* = 2000). The data collected from the online survey included information about job stressors, menstruation symptoms and psychological distress. The Graduate School of Medicine and Faculty of Medicine research ethics committee of The University of Tokyo approved the study protocol (No. 2023058NI‐(1)). This paper was reported according to the Strengthening the Reporting of Observational Studies in Epidemiology (STROBE) guidelines for reporting observational studies (Data S1).

### 2.2 Participants

Participants were recruited from the registered panel members of an Internet survey company (Cross Marketing Inc.; https://www.cross‐m.co.jp/en/). The total number of the registered panel was over 5.6 million and comprised individuals with a wide range of sociodemographic backgrounds to be nationally representative. The participants' eligibility criteria in this study were: (1) Japanese women, (2) aged from 20 to 44 years old, (3) never pregnant, (4) full‐time employees, and (5) not taking contraceptives during the past year. There were no exclusion criteria. Potential participants were invited via email based on the registered information about sex and age. Participants who consented to the study participation after reading the detailed study information and met eligibility criteria answered the questionnaire. The panellists had the options of not responding to any part of the questionnaire and discontinuing the survey at any point. The survey was closed when the target number of respondents was reached.

### 2.3 Measurement Variables

#### 2.3.1 Menstruation‐Related Symptoms

The Menstrual Distress Questionnaire (MDQ) assessed menstruation‐related symptoms (both premenstrual and menstrual) [13]. Out of the eight subscales in MDQ, we selected six subscales (i.e., pain, concentration, behavioural change, autonomic reaction, water retention and negative affect). We excluded two subscales (i.e., arousal, control) considered less prevalent among Japanese women [14]. Participants were asked to answer questions about their symptoms, 35 each for two phases: premenstrual and during menstrual. The premenstrual period was defined as from 1 week before until the day before starting menstruation. Responses were scored using a 6‐point Likert scale ranging from 1 (no reaction at all) to 6 (acute or partially disabling), with higher scores indicating a greater severity of perimenstrual and menstrual symptoms. The Japanese version of MDQ was tested for its validity and reliability [15].

In this study, menstruation‐related symptoms were calculated by summing the perimenstrual MDQ scores (range: 35–210) and those during menstruation (range: 35–210), resulting in a total score range of 70–420. A previous study using a large number of Japanese women (*n* = 19 254) reported that a score under 102 was categorised as moderate or low severity [2]. This study thus used the cut‐off point to determine the existence of severe menstruation‐related symptoms as over 103.

#### 2.3.2 Job Stressors

Job stressors were assessed based on the Job‐Demand‐Control (JDC) model and the Job‐Demand‐Control‐Support (JDCS) model [1, 16]. Four components of the JDC/JDCS model, job demands (quantitative job overload), job control, coworker support, and supervisor support, were measured using the corresponding subscales of the Brief Job Stress Questionnaire (BJSQ) [17, 18]. Each scale comprised three items, with each being rated on a 4‐point Likert‐type scale from ‘very much so’ = 1 to ‘not at all’ = 4 for job demands and job control and from ‘Extremely’ = 4 to ‘not at all’ = 1 for supervisor and coworker support. Total scores for each subscale ranged from 3 to 12, with a higher score indicating the higher degree of the corresponding component. The Japanese version of BJSQ was tested for its validity and reliability [19]. This study categorised each of four job stressors as high or low, using the cut‐off as a mean score.

#### 2.3.3 Psychological Distress

Psychological distress was measured by the Kessler 6 psychological distress scale (K6) [20, 21]. This scale contains six items assessing nervousness, hopelessness, restlessness or fidgeting, feelings of worthlessness, sadness, and perception that everything requires great effort, as experienced in the past 4 weeks, on a 5‐point Likert scale. The total score was calculated by summing all items (range: 0–24). Higher scores imply higher distress. A score of more than 5 is indicative of having distress [22, 23]. The Japanese version of K6 was tested for its validity and reliability [21].

#### 2.3.4 Covariates

##### 2.3.4.1 Demographic Variables

Age, marital status (unmarried, married, bereaved/divorced), educational attainment (junior high/high school, technology/vocational/junior college, university, graduate school), and household income (less than 2 million yen, 2–3.99, 4–5.99, 6–7.99, 8–9.99, over 10 million yen) were asked as demographic variables. Age was used as a continuous variable. The other variables were dichotomized after determining reference groups: marital status (unmarried [ref] and others), educational attainment (less than university [ref] and university/graduate school), household income (less than 4 million yen [ref] and 4 million yen or more).

##### 2.3.4.2 Working Hours and Night Shift Work

The number of monthly working hours was asked and initially treated as a continuous variable. Subsequently, this information was converted into a dichotomized variable by calculating the weekly working hours and categorising them as less than 40 h per week [ref] and 40 h or more. To determine whether participants had night shifts, they were asked, ‘Do you work night shifts (between 10:00 PM and 5:00 AM) at least three times per month?’ and were instructed to answer with either ‘Yes’ or ‘No’ [24].

##### 2.3.4.3 Hospital Visits History

A history of hospital visits due to psychiatric illness and gynaecological illness were also used as covariates. A single question assessed visits due to psychiatric illness, ‘Have you ever visited a psychosomatic medicine or psychiatry clinic?’ Visits due to gynaecological illness were assessed by being diagnosed by a doctor with any of eight gynaecological diseases: (1) uterine fibroid, (2) endometriosis, (3) uterine adenomyosis, (4) premenstrual symptoms (PMS), (5) benign ovarian tumour, (6) polycystic ovary syndrome (PCOS), (7) malignant tumours (e.g., cervical cancer, endometrial cancer, ovarian tumour), (8) others. We determined their visits if the participants reported any of these experiences.

### 2.4 Management of Data Quality

To validate data quality, we excluded respondents showing discrepancies or artificial/unnatural responses. One item (‘Please choose the second item from the bottom’) was used to detect discrepancies.

### 2.5 Statistical Analysis

Demographic variables were summarised for the participants, stratified by the levels of job stressors. Before the examination, confirmatory factor analyses were performed to ensure the independence of the constructs related to menstrual symptoms and psychological distress. We also compared the model fit indices of our hypothesized model, which posited psychological distress as a mediator, with an alternative model that considered menstruation‐related symptoms as a mediator. The models were estimated using the maximum likelihood method within a structural equation modelling framework.

For the preliminary analysis of the examination, logistic regression analyses were conducted on the menstruation‐related symptoms (i.e., MDQ ≥ 103) and psychological distress (i.e., K6 ≥ 5). Also, *E*‐values were calculated to discuss the potential effects of unmeasured confounding [25]. In addition, the interaction effects of psychological distress on the association between job stressors and menstruation‐related symptoms were were examined using methods proposed in a previous article [26]. In these analyses, we estimated the odds ratios (ORs) for job stressors within each stratum of psychological distress, and calculated both additive (relative excess risk due to interaction, RERI) and multiplicative scales.

For the main analysis, four‐way decomposition analysis [12] was used to estimate the relative contributions of psychological distress (mediation and interaction) to the potential pathways from job stressors and menstruation symptoms. We assessed the extent to which the total effect of exposure (job stressors) and outcomes (menstruation‐related symptoms) was explained by alternative pathways involving mediators (mental health). Four components of controlled direct effect (CDE), reference interaction effect (INT_ref_), mediated interaction effect (INT_med_) and pure indirect effect (PIE) decomposed the associations [27]. The causal diagram of these four pathways is illustrated in Figure 1. Studies in epidemiology using the same methodology are available elsewhere [28, 29, 30]. CDE is the effect of job stressors on menstruation‐related symptoms when fixing psychological distress at a low level (i.e., K6 < 5) for all the participants. INT_ref_ is the effect due only to the interaction between job stressors and psychological distress. INT_med_ is the effect that is due to both interactions between job stressors and psychological distress and the fact that job stressors cause psychological distress. PIE is the effect due only to mediation through psychological distress, not accounting for the possible interaction between job stressors and psychological distress. To estimate the four decomposed effects, we used the *med4way* package in Stata version 18 [12]. We adopted the logistic regression model for both the outcome (i.e., MDQ ≥ 103) and mediator (i.e., K6 ≥ 5). The med4way package estimated the four effects when psychological distress was at a low level (i.e., K6 < 5). For the covariates, age was set to the mean value (i.e., 34), and the other categorical covariates were set to the reference group (i.e., 0). The 95% confidence intervals of the effects were estimated by the default methods of the package, bootstrapping with 1000 resampling. The level of significance was set at *p* < 0.05 in all analyses. After estimating the four decomposed effects, we calculated the proportion of the total effect. Also, we described the proportion attributable to interaction (INTref + INTmed), the proportion eliminated (proportion of the effect would be eliminated when if setting everyone's psychological distress to at a low level, INTref + INTmed + PIE), and the proportion mediated (INTmed + PIE).

There were no missing values because the participants could not complete the online survey when they had even one unanswered item. Therefore, we did not adopt any methods to consider potential bias due to missing data loss.

Stata/SE version 18 was used for all analyses conducted in the study.

## 3 Results

Of a total of 2000 respondents, 182 (9.1%) participants answered incorrectly and were excluded from this process. The remaining 1818 working women without a pregnancy experience were analysed. Table 1 shows the demographic characteristics of the study participants and outcomes by job stressors (high or low). The mean age was 33.8 (standard deviation; 6.4). Unmarried (80.3%) and graduate degree or over (61.4%) comprise the majority. Participants with psychological distress (K6 more than 5) comprised 54.1%, and 54.7% showed severe menstruation‐related symptoms.

**TABLE 1:** Demographic characteristics (*N* = 1818).

	Total (*N* = 1818)	Job demands		Job control		Supervisor support		Coworker support	
		High (*N* = 1025)	Low (*N* = 793)	High (*N* = 974)	Low (*N* = 844)	High (*N* = 730)	Low (*N* = 1088)	High (*N* = 724)	Low (*N* = 1094)
	*n* (%)	*n* (%)	*n* (%)	*n* (%)	*n* (%)	*n* (%)	*n* (%)	*n* (%)	*n* (%)
Age, mean (SD)	33.76 (6.4)	33.78 (6.5)	33.73 (6.4)	33.73 (6.4)	33.78 (6.4)	33.14 (6.5)	34.17 (6.3)	32.96 (6.6)	34.28 (6.3)
Marital status									
Unmarried	1455 (80.3)	820 (80.0)	635 (80.1)	783 (80.4)	672 (79.6)	567 (77.7)	888 (81.6)	557 (76.9)	898 (82.1)
Married	318 (17.5)	176 (17.2)	142 (17.9)	166 (17.0)	152 (18.0)	141 (19.3)	177 (16.3)	151 (20.9)	167 (15.3)
Bereaved or divorced	45 (2.5)	29 (2.8)	16 (2.0)	25 (2.6)	20 (2.4)	22 (3.0)	23 (2.1)	16 (2.2)	29 (2.7)
Educational attainment									
Junior high/high school	255 (14.0)	130 (12.7)	125 (15.8)	126 (12.9)	129 (15.3)	92 (12.6)	163 (15.0)	94 (13.0)	161 (14.7)
Technology/vocational/junior college	446 (24.5)	263 (25.7)	183 (23.1)	200 (20.5)	246 (29.2)	162 (22.2)	284 (26.1)	169 (23.3)	277 (25.3)
University	1040 (57.2)	576 (56.2)	464 (58.5)	593 (60.9)	447 (53.0)	437 (59.9)	603 (55.4)	421 (58.2)	619 (56.6)
Graduate school	77 (4.2)	56 (5.5)	21 (2.7)	55 (5.7)	22 (2.6)	39 (5.3)	38 (3.5)	40 (5.5)	37 (3.4)
Household income (JPY)									
200 >	93 (5.1)	43 (4.2)	50 (6.3)	44 (4.5)	49 (5.8)	28 (3.8)	65 (6.0)	29 (4.0)	64 (5.9)
200–< 400	472 (26.0)	251 (24.5)	221 (27.9)	245 (25.2)	227 (26.9)	178 (24.4)	294 (27.0)	168 (23.2)	304 (27.8)
400–< 600	467 (25.7)	258 (25.2)	209 (26.4)	257 (26.4)	210 (24.9)	171 (23.4)	296 (27.2)	165 (22.8)	302 (27.6)
600–< 800	318 (17.5)	193 (18.8)	125 (15.8)	169 (17.4)	149 (17.7)	134 (18.4)	184 (16.9)	133 (18.4)	185 (16.9)
800–< 1000	215 (11.8)	129 (12.6)	86 (10.8)	120 (12.3)	95 (11.3)	100 (13.7)	115 (10.6)	99 (13.7)	116 (10.6)
≥ 1000	253 (13.9)	151 (14.7)	102 (12.9)	139 (14.3)	114 (13.5)	119 (16.3)	134 (12.3)	130 (18.0)	123 (11.2)
Psychiatric illness history									
No	1484 (81.6)	830 (81.0)	654 (82.5)	818 (84.0)	666 (78.9)	604 (82.7)	880 (80.9)	603 (83.3)	881 (80.5)
Yes	334 (18.4)	195 (19.0)	139 (17.5)	156 (16.0)	178 (21.1)	126 (17.3)	208 (19.1)	121 (16.7)	213 (19.5)
Gynaecological illness history									
No	1501 (82.6)	844 (82.3)	657 (82.9)	817 (83.4)	684 (81.0)	603 (82.6)	898 (82.5)	605 (83.6)	896 (81.9)
Yes	317 (17.4)	181 (17.7)	136 (17.2)	157 (16.1)	160 (19.0)	127 (17.4)	190 (17.5)	119 (16.4)	198 (18.1)
Night‐shift work (22:00–5:00)									
No	1640 (90.2)	886 (86.4)	754 (95.1)	907 (93.1)	733 (86.9)	664 (91.0)	976 (89.7)	657 (90.8)	983 (89.9)
Yes (≥ 3 days per month)	178 (9.8)	139 (13.6)	39 (4.9)	67 (6.9)	111 (13.2)	66 (9.0)	112 (10.3)	67 (9.3)	111 (10.2)
Working hours (per week)									
< 40 h	1256 (69.1)	592 (57.8)	664 (83.7)	678 (69.6)	578 (68.5)	506 (69.3)	750 (68.9)	491 (67.8)	765 (69.9)
≥ 40 h	562 (30.9)	433 (42.2)	129 (16.3)	296 (30.4)	266 (31.5)	224 (30.7)	338 (31.1)	233 (32.2)	329 (30.1)
Psychological distress									
K6 < 5	834 (45.9)	411 (40.1)	423 (53.3)	501 (51.4)	333 (39.5)	411 (56.3)	423 (38.9)	425 (58.7)	409 (37.4)
K6 ≥ 5	984 (54.1)	614 (59.9)	370 (46.7)	473 (48.6)	511 (60.6)	319 (43.7)	665 (61.1)	299 (41.3)	685 (62.6)
Menstruation‐related symptoms									
MDQ < 103	823 (45.3)	417 (40.7)	406 (51.2)	457 (46.9)	366 (43.4)	346 (47.4)	477 (43.8)	362 (50.0)	461 (42.1)
MDQ ≥ 103	995 (54.7)	608 (59.3)	387 (48.8)	517 (53.1)	478 (56.6)	384 (52.6)	611 (56.2)	362 (50.0)	633 (57.9)

The confirmatory factor analysis confirmed that psychological distress was conceptually distinct from menstruation‐related symptoms. This conclusion was based on the superior fit indices of the models assuming distinct factors compared to those assuming a single‐order factor, both in the pre‐menstruation phase (*χ* ^2^ [1] = 6296.58, *p* < 0.001) and during menstruation (*χ* ^2^ [1] = 6480.73, *p* < 0.001). Furthermore, the model fit for the alternative model, which considered menstruation‐related symptoms as a mediator, was inferior to that of our hypothesized model (CFI = 0.743, TLI = 0.422, RMSEA = 0.171, SRMR = 0.082), which posited psychological distress as a mediator (CFI = 0.987, TLI = 0.971, RMSEA = 0.038, SRMR = 0.020).

Table 2 shows the associations of job‐related stressors and mental health with menstruation‐related symptoms. Of the four kinds of job stressors, only high job demands (OR = 1.50, 95% CI, 1.22–1.83, < 0.001) and low coworker support (OR = 1.57, 95% CI, 1.22–2.02, < 0.001) showed significant positive associations with menstruation‐related symptoms in the adjusted model (Model 4). *E*‐values for these associations were 2.37 for job demands and 2.52 for coworker support. We further examined the interaction effects of psychological distress on the association between job stressors and menstruation‐related symptoms (Tables 3 and 4). The ORs tended to increase when both job stressors and psychological distress existed. However, ORs within strata of psychological distress were not much different between low distress (i.e., K6 < 5) and high distress (i.e., K6 ≥ 5). The ratio of ORs between the strata was 0.86 (95% CI, 0.69–1.03, *p* = 0.109) for job demands and 0.99 (95% CI, 0.78–1.19, *p* = 0.897) for coworker support. RERI was 0.29 (95% CI, −1.06–1.65, *p* = 0.671) for job demands and 1.04 (95% CI, 0.34–2.41, *p* = 0.139) for coworker support.

**TABLE 2:** Associations of job stressors and psychological distress with menstruation‐related symptoms (*N* = 1818).

Independent variables	Dependent variables									
	Psychological distress (K6 ≥ 5)				Menstruation‐related symptoms (MDQ ≥ 103)					
	Model 1—Crude model^a^		Model 2—Adjusted model^b^		Model 3—Crude model^a^		Model 4—Adjusted model 1^b^		Model 5—Adjusted model 2^b^	
	OR (95% CI)	*p*	OR (95% CI)	*p*	OR (95% CI)	*p*	OR (95% CI)	*p*	OR (95% CI)	*p*
Job demands (high)	1.71 (1.42–2.06)	< 0.001	1.88 (1.52–2.32)	< 0.001	1.53 (1.27–1.84)	< 0.001	1.50 (1.22–1.83)	< 0.001	1.26 (1.02–1.57)	0.034
Job control (low)	1.63 (1.35–1.96)	< 0.001	1.28 (1.04–1.56)	0.019	1.15 (0.96–1.39)	0.129	1.01 (0.83–1.23)	0.933	0.93 (0.76–1.15)	0.512
Supervisor support (low)	2.03 (1.67–2.45)	< 0.001	1.20 (0.94–1.55)	0.150	1.15 (0.96–1.39)	0.136	0.87 (0.68–1.12)	0.295	0.80 (0.62–1.06)	0.119
Coworker support (low)	2.38 (1.96–2.88)	< 0.001	2.17 (1.69–2.80)	< 0.001	1.37 (1.14–1.66)	0.001	1.57 (1.22–2.02)	< 0.001	1.26 (0.97–1.64)	0.088
Psychological distress (K6 ≥ 5)					4.37 (3.59–5.32)	< 0.001			3.87 (3.14–4.78)	< 0.001

^a^ ORs in the crude models indicated bivariate associations between independent and dependent variables.

^b^ In the adjusted models, independent variables and covariates (age, marital status, educational status, household income, and psychiatric and gynaecological illness history, night‐shift work, and working hours) were entered simultaneously.

**TABLE 3:** Analyses of the modification effect of psychological distress on the association between job demands and menstruation‐related symptoms (*N* = 1818).

	Job demands: Low		Job demands: High		ORs (95% CI) within strata of psychological distress
	*N* with/without menstrual symptoms	OR (95% CI)	*N* with/without menstrual symptoms	OR (95% CI)	
Psychological distress					
K6 < 5	133/423	1.0 (reference)	165/411	1.38 (1.02–1.87) *p* = 0.035	1.35 (0.98–1.86) *p* = 0.065
K6 ≥ 5	254/370	4.27 (3.13–5.83) *p* < 0.001	443/614	4.95 (3.72–6.59) *p* < 0.001	1.17 (0.69–1.24) *p* = 0.289

*Note:* Measure of effect modification on additive scale: RERI (95% CI) = 0.29 (−1.06 to 1.65): *p* = 0.671. Measure of effect modification on multiplicative scale: ratio of ORs (95% CI) = 0.86 (0.69–1.03); *p* = 0.109. ORs were adjusted for job control, supervisor support, coworker support, age, marital status, educational status, household income, psychiatric and gynaecological illness history, night‐shift work, and working hours.

**TABLE 4:** Analyses of the modification effect of psychological distress on the association between coworker support and menstruation‐related symptoms (*N* = 1818).

	Coworker support: High		Coworker support: Low		ORs (95% CI) within strata of psychological distress
	*N* with/without menstrual symptoms	OR (95% CI)	*N* with/without menstrual symptoms	OR (95% CI)	
Psychological distress					
K6 < 5	154/425	1.0 (reference)	144/409	1.19 (0.85–1.66) *p* = 0.315	1.33 (0.91–1.96) *p* = 0.140
K6 ≥ 5	208/299	3.60 (2.60–4.99) *p* < 0.001	489/685	4.83 (3.50–6.65) *p* < 0.001	1.23 (0.84–1.78) *p* = 0.286

*Note:* Measure of effect modification on additive scale: RERI (95% CI) = 1.04 (−0.34 to 2.41): *p* = 0.139. Measure of effect modification on multiplicative scale: ratio of ORs (95% CI) = 0.99 (0.78–1.19); *p* = 0.897. ORs were adjusted for job demands, job control, supervisor support, age, marital status, educational status, household income, psychiatric and gynaecological illness history, night‐shift work, and working hours.

Table 5 shows the decomposition of the estimated effects. Regarding high job demands, the total effect was significant (coefficient = 0.49, 95% CI, 0.13–0.85, *p* = 0.007). In the four‐way decomposition model, the PIE accounted for the largest part (48.4%), and CDE was the second largest part (37.8%) of the total effect. In contrast, the INT_ref_ and INT_med_ did not account for the significant proportions of the total effects. Regarding coworker support, the total effect on menstruation‐related symptoms was larger than that of job demands (coefficient = 0.65, 95% CI, 0.17–1.13, *p* = 0.007). In this association, the PIE accounted for the largest part (40.3%). Effects by interaction were also certain (44.1%). The CDE was little and insignificant (15.6%).

**TABLE 5:** Four‐way decomposition model.

	High job demands × Psychological distress			Low coworker support × Psychological distress		
	Estimated effect (95% CI)	*p*	Proportion attributable (95% CI)	Estimated effect (95% CI)	*p*	Proportion attributable (95% CI)
Total effect	0.49 (0.13–0.85)	0.007	N/A	0.65 (0.17–1.13)	0.007	N/A
CDE	0.19 (−0.00 to 0.34)	0.054	37.8% (3.6–72.0)	0.10 (−0.11 to 0.31)	0.339	15.6% (−11.6 to 42.8)
INT_ref_	0.05 (−0.17 to 0.27)	0.679	9.4% (−30.2 to 49.1)	0.18 (−0.08 to 0.44)	0.173	28.0% (1.7–54.3)
INT_med_	0.02 (−0.08 to 0.12)	0.680	4.4% (−13.9 to 22.6)	0.10 (−0.05 to 0.26)	0.185	16.0% (1.3–30.8)
PIE	0.24 (0.14–0.34)	< 0.001	48.4% (10.1–86.7)	0.26 (0.15–0.38)	< 0.001	40.3% (7.4–73.2)
Proportion attributable to interaction (INT_ref_ + INT_med_)			13.8% (−44.1 to 71.7)			44.1% (3.9–84.2)
Proportion eliminated (INT_ref_ + INT_med_ + PIE)			62.1% (28.0–96.4)			84.4% (57.2–111.6)
Proportion mediated (INT_med_ + PIE)			52.7% (26.9–78.6)			56.4% (31.0–81.7)

*Note:* Each model was adjusted by the other job stressors and the covariates (age, marital status, educational status, household income, and psychiatric and gynaecological illness history, night‐shift work, and working hours).

Abbreviations: CDE, controlled direct effect; INT_med_, mediated interaction; INT_ref_, reference interaction; PIE, pure indirect effect.

## 4 Discussion

### 4.1 Main Findings

This study investigated the underlying pathways linking job stressors and menstruation‐related symptoms, with a focus on the mediation and interaction of psychological distress using a cohort of pre‐conception working women. This study found that the total effect of job stressors on menstruation‐related symptoms was observed only for high job demands and low coworker support, with approximately 50% higher risk of menstruation‐related symptoms. Therefore, improving job stressors, especially job demands and coworker support, is potentially important to prevent menstruation‐related symptoms. The interaction effects of psychological distress on these associations potentially exist since both PERIs were positive, particularly assumed on the effect of coworker support. However, since the ratio of ORs is below 1, the clear modifying effect of psychological distress was not supported.

Among the total effects of the 4‐way decomposition model, mediating effects were obvious: effects of high psychological distress associated with high job demands (PIE, 48.4%) and low coworker support (PIE, 40.3%). Therefore, high job demands and low coworker support can affect menstruation‐related symptoms through deteriorating mental health status. In addition, there was a difference in the decomposition between job demands and coworker support. In the case of job demands, CDE and PIE accounted for 86.2%, with the proportion of the effect attributable to the interaction with psychological distress being minimal (13.8%). In contrast, for coworker support, the effect due to interaction with psychological distress was 44.1%, suggesting that the combination of low coworker support and high psychological distress may have a substantial impact on menstrual‐related symptoms. Regarding the association with menstruation‐related symptoms, coworker support is more strongly related to poor mental health, including its mediating and moderating effects, than job demands.

### 4.2 High Job Demands and Low Coworker Support

Job demands and low coworker support were mainly associated with the outcome in the present study. This result coincided with a previous report that quantitative workload demand was associated with the severity of premenstrual symptoms [7]. Job demands can lead to additional adverse psychosocial factors, such as long working hours and physical demands (or inactivity). They may directly affect physical and mental recovery, including sleep quality, resulting in health issues. Regarding coworker support, our result also coincided with a previous report that low coworker support was associated with severe menstrual pain [31]. When coworker support is low, individuals may experience increased pressure and responsibility regarding their work, leading to a greater sense of burden. Additionally, low coworker support may make it more difficult to take time off, potentially reducing access to medical care. High job demand and low coworker support were found to be an important job stressor on menstruation‐related symptoms.

### 4.3 Mediating Effect of Psychological Distress

This study showed nearly half of the effect of the path from job demands and coworker support to menstruation‐related symptoms may be explained by the mediating effects of mental health. Mediating effects can be explained that those with high job stressors developed poor mental health from increased stress‐related hormones or decreased neurotransmitters (e.g., serotonins) [32]; poor mental health itself worsens menstruation‐related symptoms by increasing susceptibility to sex hormone fluctuation. Epidemiological studies consistently show patterns that those with poor mental health have more difficulties when they face reproductive events, such as premenstrual, pregnancy, postpartum and menopause [33, 34]. Poor mental health causes vulnerability to hormone fluctuation, partially via negative cognition or poor coping behaviour, and may lead to menstruation‐related symptoms. The mechanism of the relation between mental health and menstruation‐related may be similar to premenstrual exacerbation (PME): existing mental conditions worsen the various symptoms before menstruation. PME does not arise from abnormal hormone levels, but symptoms are triggered by abnormal sensitivity to normal fluctuations of sex hormones and their metabolites across the menstrual cycle [35]. Cycle‐related fluctuations of sex hormones also moderate stress sensitivity, which influences emotion regulation [36].

### 4.4 Interaction Effect of Psychological Distress

The effect due to interaction with psychological distress was small (13.8%) in job demands, and large in coworker support (44.1%). This means that 44% of the adverse impacts of low coworker support on menstruation outcomes can be prevented by improving mental health. Interaction effects can be explained that, if an individual has deteriorated mental health, job stressors worsen menstruation‐related symptoms, but if their mental health is good, job stressors do not have much effect on menstruation‐related symptoms. Poor mental health increases the sensitivity of job stressors to menstruation‐related symptoms by diminishing the sense of control or mastery [37]. These results suggested the importance of mental health for menstruation outcomes.

There was also a pathway (INT_med_) in which the lack of coworkers support, exacerbates psychological distress, thereby strengthening the interaction effect of psychological distress. For female workers, a lack of support from colleagues may worsen mental health and increase subjective work burden through negative cognition, activate the hypothalamic–pituitary–adrenal axis, and heighten autonomic nervous system sensitivity, potentially exacerbating menstruation‐related symptoms.

### 4.5 Job Control and Supervisor Support

This study did not show a significant association of job control and supervisor support on menstruation‐related symptoms. It was inconsistent with a previous cross‐sectional study reporting that low job control was associated with a higher risk for menstrual pain [31]. Previous Japanese cross‐sectional study also suggested that job control may be important to adopting coping behaviour towards menstruation [38]. The impact of supervisor support was not well described in previous reports, our result suggested the impact of perceived support from supervisor may relatively small. But since those study did not employ the same model as ours, which included psychological distress, it is still unsure about the associations. Further study is needed to replicate the findings.

### 4.6 Impact of Our Findings

Women worldwide are 2–3 times more likely than men to suffer from depression in their lifetime [39]. This sex difference is explained by changes in reproductive steroid hormones during reproductive transitions: the menstrual cycle, pregnancy, the peripartum and the menopause [36]. Pre‐conception health is essential to prevent mental health issues later in life. Both abnormal menstruation and poor mental health in the pre‐conception period are risk factors for perinatal depression [40, 41]. Our results showed that high job stressors and induced poor mental health caused adverse menstruation‐related symptoms, potentially suggesting the increased sensitivity of mental health on menstruation. There is growing evidence to show these biological associations between mental health and menstruation: interactions between the hypothalamic pituitary adrenal and the hypothalamic–pituitary‐gonadal axis [42], between the serotonergic and GABAergic systems [43], and sensitivity to neurosteroid allopregnanolone fluctuation [44]. Improving pre‐conception workers' mental health may minimise the impact of hormone fluctuation. This study suggested the need to pay more attention to mental health and psychosocial factors at work in clinical practice to reduce the burden of menstruation‐related symptoms. Workplace interventions, such as mental health support and flexible work arrangements, should be examined its effectiveness to alleviate female workers' health. WHO guidelines on mental health at work also recommend multilevel approaches, such as organisational interventions, which can be implemented in the workplace [45]. Appropriate intervention methods should be identified.

### 4.7 Strengths and Limitations

This study's strengths are the participants and measurements. Although symptoms relating to menstruation vary depending on the number of childbirth deliveries and contraceptive devices, this study limited participants to those who were currently working full‐time without pregnancy experience or contraceptive use. The study's strength was recruiting a large sample of pre‐conceptional female workers under similar working/biological/medical conditions. Next, the exposures to job stressors were comprehensively measured, and MDQ measured the outcome before and during menstruation (70 items in total). The data of this study was obtained under ideal conditions for the present analysis.

However, several limitations should be cautioned. First, this study had a cross‐sectional design. Mediation analysis using cross‐sectional data is not ideal due to causal reserve issues. Still, in our model, the time needed to change outcomes was unsure, and it was difficult to establish the longitudinal model using appropriate variables at a proper time. However, it was demonstrated that cross‐sectional approaches to mediation typically generate substantially biased estimates of longitudinal parameters even under the ideal conditions when mediation is complete [46, 47]. Further studies are needed to collect the basic information needed to conduct longitudinal analyses. In addition, although our study highlights the relationship between job stress and menstruation‐related symptoms, we cannot entirely rule out the possibility that women with more severe menstruation‐related symptoms may self‐select into higher‐stress jobs as a coping strategy, using job demands to distract from their symptoms. Given the cross‐sectional design of this study, we cannot adequately exclude the possibility of job selection effects, which refer to the influence of pre‐existing individual characteristics that lead people to select certain jobs, rather than the causal impact of the jobs themselves. Second, the causal meditation analysis is based on a strong set of identification assumptions, including no unmeasured confounding for the exposure‐outcome association. While we controlled for possible confounders, we could not rule out the possibility of unmeasured confounding. Additionally, the analysis assumes no mediator‐outcome confounders that are themselves influenced by the exposure (e.g., an additional mediator related to mental health). This assumption is particularly critical for ensuring the validity of the results, but it is difficult to fully verify its plausibility. Violations of this assumption could lead to biased estimates and affect the causal interpretation of our findings. Third, though not a major situation, we cannot deny the possibility that menstruation‐related symptoms alleviate job stressors because of accommodations at the workplace. Fourth, all the information was obtained by a self‐reporting questionnaire, and the outcome was not diagnosis‐based. In addition, recall and reporting biases should be noted. Fifth, the experimental and ideal sample recruited online can lead to limited generalizability. Sixth, according to the calculation of the E‐value, to fully explain the observed association solely by unmeasured confounding bias, the unmeasured confounders would need to have a strong association with both the exposure and the outcome, with an OR of 2.37 or higher. Adjustments have already been made for history of visits to psychiatry and gynaecology clinics, making the likelihood of unconsidered factors low. However, factors such as genetic inheritance (e.g., parental medical history), healthcare knowledge/skills, or personality traits prone to overreporting may still exist.

### 4.8 Practical Implications

This study showed that improving mental health and reducing job demands and improve coworker support may benefit menstruation‐related symptoms of pre‐conceptional female workers. Further studies are needed to identify the time for mental health to affect menstruation‐related symptoms and to examine the relationship by using longitudinal data. In the future, an intervention of approaching both psychosocial factors at work and mental health to improve menstruation‐related symptoms should be tested.

## Author Contributions

N.S. was in charge of this study. N.S. and K.W. organised the study design and analysed the data. N.S. wrote the first draft. All other collaborators ensured that questions related to the accuracy or integrity of any part of the work were appropriately investigated and resolved. All authors participated in conducting the survey. All other authors revised it critically. All authors approved the final version of the manuscript.

## Ethics Statement

This study was approved by the Research Ethics Committee of the Graduate School of Medicine/Faculty of Medicine, The University of Tokyo, No. 2023058NI‐(1).

## Consent

Online informed consent was obtained from all participants, who were given full disclosure and explanation of the purpose and procedures of this study. We explained that their participation was voluntary, and they could withdraw consent for any reason simply by not completing the questionnaire.

## Conflicts of Interest

N.S. received personal fees from Medilio Co. Ltd., outside the submitted work. D.N. reports personal fees from Startia Inc., MD.net, and an honorarium from Takeda Pharmaceutical Co., Otsuka Medical Devices Co. Ltd. outside the submitted work. The other authors declare no conflicts of interest.

## Supporting information


Data S1.

## Data Availability

The data that support the findings of this study are available from the corresponding author upon reasonable request.
